# The Willingness of Elderly Taiwanese Individuals to Accept COVID-19 Vaccines after the First Local Outbreak

**DOI:** 10.3390/vaccines10040520

**Published:** 2022-03-27

**Authors:** Chia-Yu Huang, Ching-Chan Lin, Ching-Yun Hsieh, Chen-Yuan Lin, Tzu-Ting Chen, Pei-Ching Wu, Dung-Huan Liu, Sio-Ian Tou, Hung-Rong Yen

**Affiliations:** 1 Department of Family Medicine, Taichung Tzu Chi Hospital, Buddhist Tzu Chi Medical Foundation, Taichung 427, Taiwan; tc1811201@tzuchi.com.tw; 2 Graduate Institute of Chinese Medicine, School of Chinese Medicine, College of Chinese Medicine, China Medical University, Taichung 404, Taiwan; 3 Division of Hematology and Oncology, Department of Internal Medicine, China Medical University Hospital, China Medical University, Taichung 404, Taiwan; D13256@mail.cmuh.org.tw (C.-C.L.); D7995@mail.cmuh.org.tw (C.-Y.H.); D5761@mail.cmuh.org.tw (C.-Y.L.); D14353@mail.cmuh.org.tw (T.-T.C.); 4 Division of Hematology and Oncology, Department of Internal Medicine, An Nan Hospital, China Medical University, Tainan 709, Taiwan; 5 Department of Chinese Medicine, China Medical University Hospital, Taichung 404, Taiwan; D36619@mail.cmuh.org.tw; 6 Doctoral Degree Program of Biomedical Science and Engineering, College of Biological Science and Technology, National Yang Ming Chiao Tung University, Hsinchu 300, Taiwan; D35345@mail.cmuh.org.tw; 7 Department of Physical Medicine and Rehabilitation, China Medical University Hospital, Taichung 404, Taiwan; 8 Department of Physical Therapy, Graduate Institute of Rehabilitation Science, China Medical University, Taichung 404, Taiwan; 9 Department of Pediatrics, Chung Kang Branch, Cheng-Ching General Hospital, Taichung 407, Taiwan; 10 Research Center for Traditional Chinese Medicine, Department of Medical Research, China Medical University Hospital, Taichung 404, Taiwan; 11 Research Center for Chinese Medicine, China Medical University, Taichung 404, Taiwan

**Keywords:** COVID-19, elder, questionnaire, Taiwan, vaccine, willingness

## Abstract

Vaccination is the most effective intervention to prevent infection and subsequent complications from SARS-CoV-2. Because of their multiple comorbidities, the elderly population experienced the highest number of deaths from the COVID-19 pandemic. Although in most countries, older people have top priority for COVID-19 vaccines, their actual willingness and attitudes regarding vaccination are still unclear. Thus, we conducted a cross-sectional study to investigate their willingness, attitudes, awareness, and knowledge of COVID-19 through a web-based questionnaire after the first local outbreak of COVID-19 in Taiwan. A total of 957 questionnaires were completed, and 74.9% of elderly individuals were likely to receive COVID-19 vaccines. The results from a multiple logistic regression demonstrated that older people who need to visit the outpatient department and have a high level of concern about the safety of COVID-19 vaccines are prone to having a negative willingness to accept COVID-19 vaccines. The following items related to awareness of the COVID-19 pandemic were attributed to the acceptance of COVID-19 vaccines: “understanding the risk of being infected by SARS-CoV-2”, “understanding the effectiveness of COVID-19 vaccines”, “willingness to accept the COVID-19 vaccine for protecting others”, and “safety of COVID-19 vaccines is a key factor for you to accept them”. Furthermore, a positive association between COVID-19 vaccination and attitudes toward accepting booster doses of the COVID-19 vaccine was observed. Our results show that these factors could affect the willingness of older people to accept COVID-19 vaccines and that they are important for policymakers and medical staff to develop vaccination plans during the COVID-19 pandemic.

## 1. Introduction

The COVID-19 pandemic has been ongoing for more than 2 years, and the dominant SARS-CoV-2 variant, which began as alpha and beta, has now become omicron [1]. Although novel antiviral agents, such as molnupiravir, have been produced [2], the importance of vaccination has not changed. Except for medical staff and some government employees, elderly individuals are always the priority population asked to accept the COVID-19 vaccine in most countries [3]. Because elderly individuals with several comorbidities have a very challenging clinical course after infection by SARS-CoV-2, they represented the majority of deaths in Europe and the U.S. before the availability of COVID-19 vaccines [4]. After the COVID-19 vaccines were developed, they were helpful in controlling the spread of SARS-CoV-2 variants such as alpha and beta. Then the booster dose was also recommended when the SARS-CoV-2 delta and omicron variants appeared [1,5]. However, people who do not want to accept the COVID-19 vaccine still present a hindrance to the goal of herd immunity, which also becomes increasingly difficult to achieve because new SARS-CoV-2 variants never stop developing.

In Taiwan, a local outbreak was not identified until April 2021. Based on experiences from fighting severe acute respiratory syndrome (SARS) in 2003, the Taiwanese government executed restricted border control and home isolation to prevent people with suspected cases of SARS-CoV-2 from entering the community. Medical mask rationing, body temperature monitoring, and screening of travel history, occupation, contact history, and cluster (TOCC) before allowing people to enter public areas were performed to lower the possibility of spreading the virus within the community [4]. Although the effect of vaccination on controlling the spread of COVID-19 in the community has been noted in other countries [6], the need for COVID-19 vaccines in Taiwan was still a controversial topic at that time. Furthermore, the shortage of reliable vaccines was another obstacle to starting the vaccination plan. The source of the first case of the first local outbreak in Taiwan was difficult to trace, and this was the first time the Taiwanese faced a real threat from SARS-CoV-2. The preparations for the vaccination plans became emergent actions. People started to care about each detail of the COVID-19 vaccine that they could be ordered to receive. In clinical practice, we found that side effects of the COVID-19 vaccine, such as thrombosis, could be the major concerns in older people that cause vaccine hesitancy [7].

Studies that determine the willingness of older populations to be vaccinated are rare, and most studies do not focus on specific populations. The most common cohort included in research on vaccine willingness is medical care providers. Furthermore, the percentage of elderly participants in these studies has always been less than 10% [8]. Because the priority of vaccination in older people in most countries is high, the real attitude toward vaccination could be easily ignored. The possible factors that affect elderly individuals’ acceptance of the COVID-19 vaccine have always been ignored in previous studies. Because information on the acceptance of the COVID-19 vaccine in Taiwanese older adults is limited, we conducted a study to disclose the willingness of elderly Taiwanese individuals to receive COVID-19 vaccines after the first local COVID-19 outbreak through an online questionnaire with reference to real-world data on the acceptance rate. In addition, the factors affecting their acceptance of vaccines were also analyzed. The results will be useful for designing and performing further vaccination plans to confirm safety in the community.

## 2. Materials and Methods

### 2.1. Study Design and Sample

We designed a cross-sectional study through an online questionnaire to evaluate the willingness to receive the COVID-19 vaccination in older people from 1 June to 31 September 2021. The questionnaire was designed based on previous reports with acceptable validity of willingness to receive COVID-19 vaccination [9,10]. At the beginning of the questionnaire, the age of the participants was confirmed, and those who responded that their age < 60 years old were not allowed to finish the following parts. Vaccination status was also confirmed, and we included only those who had not received COVID-19 vaccines at the time when they responded to the questionnaire to prevent sampling bias. A total of the following 4 parts were included in the questionnaire: “basic characteristics of the participants”, “awareness of the COVID-19 pandemic and attitudes toward receiving COVID-19 vaccines”, “possible factors affecting the willingness of elderly individuals to accept COVID-19 vaccines” and “knowledge of the COVID-19 pandemic and attitudes toward accepting booster doses of COVID-19 vaccines” [11,12,13,14,15,16]. The questionnaire was pretested for face validity by a panel of three physicians who had experience in preventive medicine at Tzu Chi Hospital, China Medical University Hospital, and Cheng-Ching General Hospital. Then, six experts and scholars with different backgrounds in geriatrics, public health, medicine, immunology, and bioethics were invited to test the content validity of the questionnaire. The items in parts 2–4 were verified for their accuracy and degree of difficulty by these experts. We processed the reliability analysis to test for internal consistency. The Cronbach’s alpha coefficients were 0.82 for all items in the questionnaire, except for the item “awareness of the COVID-19 pandemic and attitudes toward receiving COVID-19 vaccines”, with a value of 0.80; the item “possible factors affecting the willingness of elderly individuals to accept COVID-19 vaccines”, with a value of 0.71; the items “knowledge of the COVID-19 pandemic and attitudes toward accepting booster doses of COVID-19 vaccines”, with a value of 0.72.

The larger the sample size is, the higher the external validity and the greater the generalizability [17]. The most recent statistics on the number of Taiwanese elderly individuals were 3,800,000 [18]. We used a sample size calculator to determine the necessary sample size to achieve sufficient statistical power, and a total of 600 elderly individuals were needed for a margin of error of ±4%, a confidence level of 95%, and a 50% response distribution [19]. The simplified snowball sampling technique was used according to previous studies for the recruitment of participants by asking the researchers to send invitations to the older people they were in contact with [20]. Snowball sampling had the advantage of being able to recruit participants during the COVID-19 pandemic, when face-to-face research was difficult to achieve [21]. To prevent sampling bias, we started to recruit participants from different sites as much as possible: hospitals (three medical centers and regional hospitals), nursing homes, and communities (23 districts). When people received the link to the questionnaire, the aim of the study, the rights of the participants, and the anonymity statement were shown in the first paragraph. This study was approved by the Ethics Committee of China Medical University Hospital, Taichung, Taiwan (CMUH110-REC3-118).

### 2.2. Variables

The basic characteristics of the participants included sex (female or male), age (60–69, 70–79, 80–89, or ≥90 years old), residential area (community, nursing home, or outpatient department), Charlson Comorbidity Index score (CCI: ≤4 or ≥5), history of influenza vaccination (yearly, not yearly, or never), preference of origin of influenza vaccine (nil, Taiwan, or overseas), and history of pneumococcal vaccination (yes or no) [4,22,23].

We evaluated the willingness of participants to receive COVID-19 vaccines by having them select one of the following five options: very unlikely, somewhat unlikely, somewhat likely, very likely, and unsure. Then, those who responded “very likely” or “somewhat likely” were defined as having a positive willingness to accept COVID-19 vaccines. The others were considered to have negative willingness to accept COVID-19 vaccines [9,24].

The COVID-19 Snapshot Monitoring Questionnaire was developed and validated to monitor public perceptions of COVID-19, and we referenced it to assess our evaluation. The questions in the section “awareness of the COVID-19 pandemic and attitudes toward receiving COVID-19 vaccines” included “understanding the risk of being infected by SARS-CoV-2”, “understanding the severity of the COVID-19 pandemic”, “understanding the effectiveness of COVID-19 vaccines”, “understanding the complications of COVID-19 vaccines”, “willingness to accept the COVID-19 vaccine for protecting others”, and “safety of COVID-19 vaccines is a key factor for you to accept them” [11,12,13,14,15,16].

The items for “possible factors that affect the willingness of elderly individuals to accept COVID-19 vaccines” included the “availability of vaccination sites”, “recommendation by family doctors”, “experiences from politicians” and “daily news from the media” [23]. We also assessed “knowledge of the COVID-19 pandemic and attitudes toward accepting booster doses of COVID-19 vaccines” in the participants. All the above questions were scored from 1–7, with scores of 1–3, 4, and 5–7 defined as low, middle, or high, respectively [9].

### 2.3. Statistical Analysis

We used the chi-square test to compare participants who had a positive willingness to accept COVID-19 vaccines with those who did not in terms of “basic characteristics of participants”, “awareness of the COVID-19 pandemic and attitudes toward receiving COVID-19 vaccines”, “the possible factors affecting the willingness of elderly individuals to accept COVID-19 vaccines” and “knowledge of the COVID-19 pandemic and attitude toward receiving booster doses of COVID-19 vaccines”. The effect size was presented by the coefficient of contingency (COC) [25]. Multiple logistic regression analysis was used to analyze the association between a positive willingness to accept COVID-19 vaccines and each variable.

In Model 1, the independent variables were sex, age, residential area, CCI, history of influenza vaccination, preference of origin of influenza vaccine, and history of pneumococcal vaccination. In Model 2, the independent variables were the variables in Model 1, plus ”understanding the risk of being infected by SARS-CoV-2”, ”understanding the severity of the COVID-19 pandemic”, ”understanding the effectiveness of COVID-19 vaccines”, ”understanding the complications of COVID-19 vaccines”, “willingness to accept the COVID-19 vaccine for protecting others”, and ” safety of COVID-19 vaccines is a key factor for you to accept them”. In Model 3, the independent variables were the variables in Model 2 plus “availability of vaccination sites”, “recommendation by family doctors”, “experiences from politicians” and “daily news from the media”. In Model 4, the independent variables were the variables in Model 3 plus “having information that SARS-CoV-2 could spread as influenza does yearly” and “willingness to accept booster doses of COVID-19 vaccines in the future”.

The commercial statistical software SPSS version 22 (IBM Corp) was used to perform the data analysis. A *p* value < 0.05 in a 2-tailed test was considered statistically significant.

## 3. Results

A total of 963 responses were collected, but incomplete data were found for 6 participants. Finally, responses from 957 participants were collected for analysis (Table 1). A total of 74.9% of participants had a positive willingness to receive COVID-19 vaccines. Those who were female (*p* = 0.008, COC = 0.086), were aged ≥ 90 years (*p* < 0.001, COC = 0.149), were nursing home residents (*p* = 0.036, COC = 0.083), had a CCI ≥ 5 (*p* = 0.002, COC = 0.099), had never accepted influenza vaccination (*p* = 0.003, COC = 0.111), or had no preference for the origin of the influenza vaccine (*p* < 0.001, COC = 0.169) had a significantly lower acceptance of COVID-19 vaccines.

**Figure 1:**
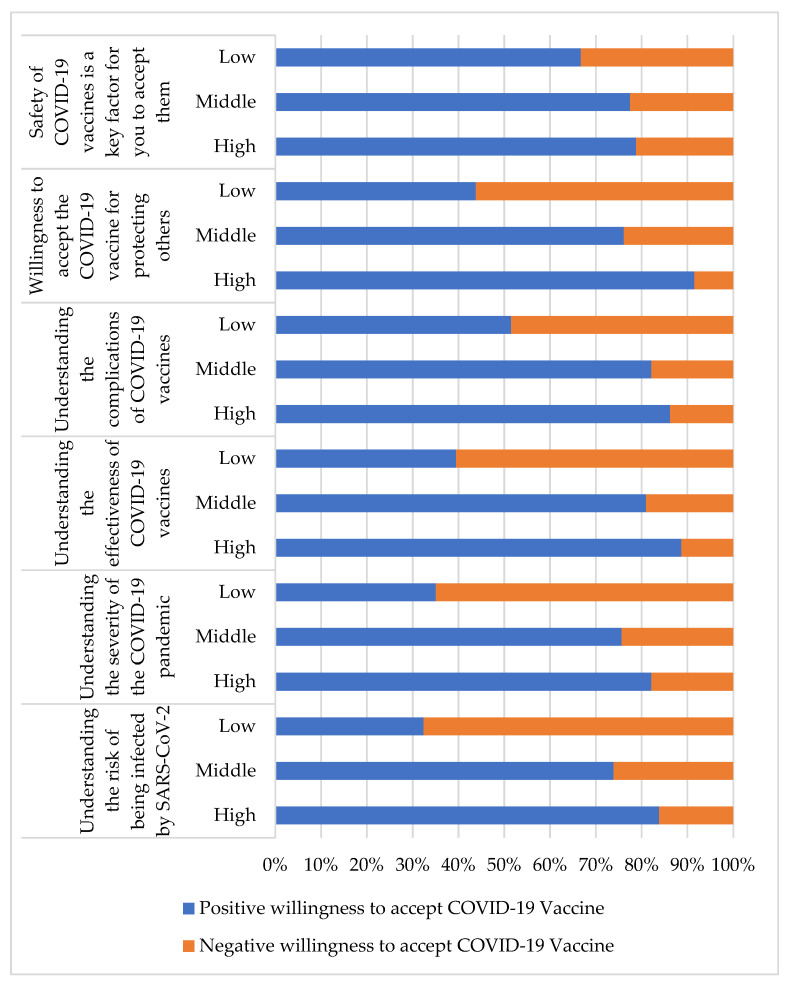
The percentages of positive and negative willingness to accept COVID-19 vaccine for each item in Table 2.

Table 2 shows the awareness of the COVID-19 pandemic and attitudes toward receiving COVID-19 vaccines. The associations between high scores in responding to the items of “understanding the risk of being infected by SARS-CoV-2” (*p* < 0.001, COC = 0.391), “understanding the severity of the COVID-19 pandemic” (*p* < 0.001, COC = 0.350), “understanding the effectiveness of COVID-19 vaccines” (*p* < 0.001, COC = 0.438), “understanding the complications of COVID-19 vaccines” (*p* < 0.001, COC = 0.340), “willingness to accept the COVID-19 vaccine for protecting others” (*p* < 0.001, COC = 0.433) and “safety of COVID-19 vaccines is a key factor for you to accept them” (*p* < 0.001, COC = 0.125) and significant positive willingness to receive the COVID-19 vaccine are shown. The percentages of participants with positive and negative willingness to accept the COVID-19 vaccine for each item are presented in Figure 1.

**Figure 2:**
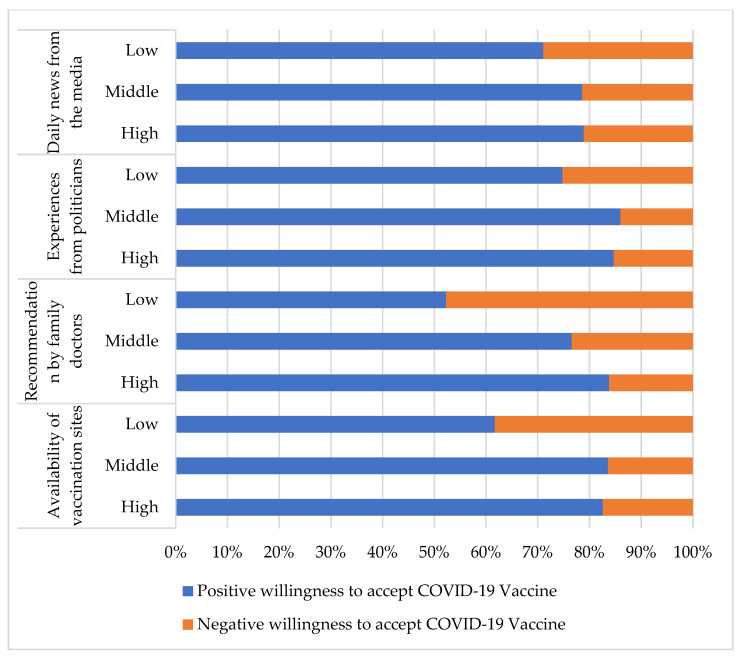
The percentages of positive and negative willingness to accept COVID-19 vaccine for each item in Table 3.

Table 3 reveals possible factors affecting the willingness of elderly individuals to accept COVID-19 vaccines. The associations between participants who had a high score for the variables of “availability of vaccination sites” (*p* < 0.001, COC = 0.231), “recommendation by family doctors” (*p* < 0.001, COC = 0.290), “experiences from politicians” (*p* < 0.001, COC = 0.201), and “daily news from the media” (*p* < 0.001, COC = 0.202) and a significant positive willingness to receive COVID-19 vaccines were noted. The percentages of participants with a positive and negative willingness to accept the COVID-19 vaccine for each item are presented in Figure 2.

**Figure 3:**
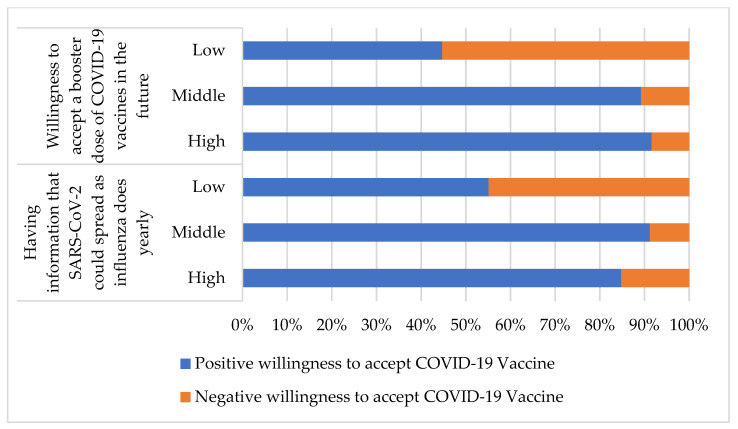
The percentages of a positive and negative willingness to accept COVID-19 vaccine for each item in Table 4.

The sections on knowledge of the COVID-19 pandemic and attitudes toward accepting a booster dose of a COVID-19 vaccine (Table 4) demonstrated the associations between a high score for the items “having information that SARS-CoV-2 could spread as influenza does yearly” (*p* < 0.001, COC = 0.330) and “willingness to accept a booster dose of COVID-19 vaccines in the future” (*p* < 0.001, COC = 0.451) and positive willingness to accept COVID-19 vaccines in elderly individuals. The percentages of participants with a positive and negative willingness to accept the COVID-19 vaccine for each item are presented in Figure 3.

The results of the multiple logistic regression analysis are shown in Table 5. In the section on the basic characteristics of participants, the age interval of 80–89 years was a significant factor for acceptance of COVID-19 vaccines (odds ratio (OR): 2.17, 95% confidence interval (CI): 1.02–4.61), but enrollment from the outpatient department was a significant factor for negative willingness to accept COVID-19 vaccines (OR: 0.47, 95% CI: 0.25–0.91). In the section on “awareness of the COVID-19 pandemic and attitudes toward receiving COVID-19 vaccines”, “understanding the risk of being infected by SARS-CoV-2” (OR: 5.19, 95% CI: 2.40–11.25), “understanding the effectiveness of COVID-19 vaccines” (OR: 6.00, 95% CI: 3.22–11.17), and “being willing to accept the COVID-19 vaccine to protect others” (OR: 8.56, 95% CI: 4.63–15.82) were significant positive factors for COVID-19 vaccination. However, “safety of COVID-19 vaccines is a key factor for you to accept them” was a significant negative factor for COVID-19 vaccination (OR: 0.54, 95% CI: 0.30–0.99).

In the section on the possible factors affecting the willingness of elderly individuals to accept COVID-19 vaccines, no item achieved statistical significance. In the section on knowledge of the COVID-19 pandemic and attitudes toward booster doses of COVID-19 vaccines, “being willing to accept booster doses of COVID-19 vaccines in the future” was a significant factor for receiving COVID-19 vaccines (OR: 5.09, 95% CI: 2.66–9.75). However, compared with participants with a low score, those with a high score for “having the information that SARS-CoV-2 could spread as influenza does yearly” were not significantly more likely to have a positive willingness to accept COVID-19 vaccines (OR: 1.01, 95% CI: 0.57–1.76).

## 4. Discussion

Our study showed the willingness of elderly Taiwanese individuals to accept COVID-19 vaccines after the first local COVID-19 outbreak in April 2021. Because of the shortage of COVID-19 vaccines, the general population in Taiwan started a vaccination plan in July 2021. However, the availability of the COVID-19 vaccine is still a problem after the following months, which has delayed the speed of vaccination. Before the first local break, the need for vaccination was discussed. Based on border control and the lack of evidence of the spread of the virus in the community, the vaccination plan was postponed. Compared to populations in other countries, few Taiwanese people had antibodies to SARS-CoV-2 infection or COVID-19 vaccination, which was a potential problem causing subsequent outbreaks. On the other hand, vaccine hesitancy in elderly Taiwanese individuals may have been caused by news reports of the deaths and severe complications after vaccination in other countries [7,26]. Although the willingness of Taiwanese people to be vaccinated has been investigated, the results from older populations have not been included or analyzed. The vaccine acceptance rate of Taiwanese individuals before the local COVID-19 outbreak was 23.4% among health care providers and 30.7% among participants (aged < 45 years) from outpatient departments [27]. Furthermore, because of restrictions and case management, there were no local cases in Taiwan at the time of the previous study. However, full vaccination is a necessary intervention in the COVID-19 pandemic to decrease hospital load, control the spread and lower the death rate, especially in the older population [7]. From the experiences of nursing home outbreaks in Europe and the U.S., the priority of the COVID-19 vaccination for elderly Taiwanese individuals was high. It is important to investigate the willingness of these older people and the factors that could affect their attitudes toward vaccination. As new variants of SARS-CoV-2 appear, ensuring full vaccination is a necessary public health plan for each country.

Our results showed that the willingness rate to be vaccinated in elderly Taiwanese individuals was 74.9%; more specifically, it was 79.8% at the age of 60–69 years, 76.8% at the age of 70–79 years, and 67.4% at the age of ≥80 years after the first local COVID-19 outbreak caused by SARS-CoV-2 variants alpha and delta. Before 2022, most Taiwanese people completed two doses of the COVID-19 vaccination of their own free will. At the end of 2021, health care providers and other populations involved in essential occupations will receive additional doses. The following actual acceptance data for elderly people were also disclosed at this time: acceptance rates were 76.6% at the age of 50–64 years, 79.7% at the age of 65–74 years, and 67.2% at the age of ≥75 years [28], which was very similar to our findings. Although many studies have tried to predict the actual acceptance rate of COVID-19 vaccinations through their investigations, our report is the first study to use real-world data to confirm the results of the investigation. Our study predicts the real-world acceptance rate after six months, which is nearly the final acceptance rate in Taiwan.

Studies investigating the willingness to receive COVID-19 vaccines in older people are rare. Most studies included elderly individuals as participants. In Europe, the willingness rate of adults in 2020 was 62–80% [29]. The latest acceptance rates for the elderly population in these countries were 88–96.93% [30,31,32,33,34,35,36]. The willingness rate in adults in the U.S. in 2020 was 65.9–86% [8,13,37], and the actual vaccination rate of the older population in 2022 was 88.40% [38]. In the Middle East, the willingness rate for COVID-19 vaccination in adults was 23.6–31.8% [39], and 21.2–93% of elderly individuals accepted vaccination, according to real-world data [40,41,42]. In Asia, the willingness rates for COVID-19 vaccination in adults in Indonesia and China in 2020 were 78.3% and 80.0%, respectively [14,43]. The actual acceptance rates of older residents were 67.4% in 2022 [44] and ≥80% at the end of 2021 [45]. A Japanese study revealed the following willingness of elderly people to receive COVID-19 vaccines: 77.2% in 2020 [46], and the actual acceptance rate was ≥90.4% at the end of 2021 [47]. In Canada, the willingness of older people to receive COVID-19 vaccines was 84.8–86% in 2020 [48], and the actual vaccination rate in early 2022 was 93.5–96.95% [49].

Clinical presentations in elderly individuals are varied and are not always consistent with the findings in adults. This situation was also noted regarding the reasons and attitudes for the acceptance of the COVID-19 vaccines. Certain basic characteristics, such as female sex, younger age, and no history of previous influenza vaccination, were the factors in the general population associated with a low willingness to receive COVID-19 vaccination [23]. Although being female and having no history of previous influenza vaccination were also found to be significant negative factors for COVID-19 vaccination in our results in Model 1, they were not found to have an influence in Model 3 or 4. The older cohort was more prone to accepting the vaccine than the younger elderly cohort, which is compatible with the findings of a previous study [48]. The older participants recruited from the outpatient department showed a negative willingness to receive the vaccine. They may be afraid of dying after vaccination, which could be a result of poor underlying disease control. The factors in the section on “awareness of the COVID-19 pandemic and attitudes toward receiving COVID-19 vaccines” are also important for elderly individuals to make decisions regarding vaccination. A previous Taiwanese study also found an association between people’s having good knowledge of COVID-19 and a positive willingness for vaccination [50].

Interestingly, the safety of the COVID-19 vaccine is a significant factor in discouraging older people from receiving the vaccine. However, as in the first local outbreak, elderly Taiwanese individuals did not rank the safety of the vaccine as a very important issue. Those who were more cautious about the safety of the vaccines were less willing to accept them. The same results were also noted in a Canadian report, as follows: the safety of COVID-19 vaccines was the major concern for elderly individuals unlikely to accept vaccines [48].

“Availability of vaccination sites”, “recommendation by family doctors”, “experiences from politicians”, and “daily news from the media” have been found to have significant relationships with the acceptance of COVID-19 vaccines in the general population [23], which is not consistent with our results. Because most of the items in previous investigations of the willingness of elderly individuals were not consistent with our designs or published studies, the factors relating to awareness of the COVID-19 pandemic are common and significant findings that could affect the acceptance of vaccination [21].

The coverage rates of COVID-19 vaccination in older populations in some Asian countries and regions are lower than those in Europe or the U.S. [14,43]. They have similar measures for border control; the necessity of vaccination may not have existed before local outbreaks. This situation would cause disaster outbreaks in communities such as Hong Kong because most older people lack antibodies to fight SARS-CoV-2 [51]. The participants in our study were Asian elderly individuals and the results are useful as a reference for those regions with low vaccination rates to design further vaccination plans and prevent future outbreaks.

Many countries give people who have accepted full COVID-19 vaccinations the certification of a “green pass”, which helps people visit any place without limitations [7]. However, the Taiwan CECC did not practice this measure, except for hospital admission or nursing home visitation. TOCC screening at hospital entrances in Taiwan has not ceased during the COVID-19 pandemic. Before Taiwan had COVID-19 vaccines, Taiwanese people were asked to offer a negative antigen rapid test result for SARS-CoV-2 before they entered a nursing home or hospital admission. This requirement could be replaced when people have the full COVID-19 vaccination for 14 days. Respecting people’s willingness for COVID-19 vaccination is the main reason why the Taiwan CECC did not execute a “green pass” approach comprehensively. It is important to know that, even after the local outbreak, the total confirmed number of local cases in Taiwan was 15,493 [52] and the total population of Taiwan was 23.57 million. The existence of a “green pass” in Taiwan is not as urgent as in other places.

There are several limitations to our study. The web-based questionnaire was administered and completed on a voluntary basis, and selection bias may exist. The willingness rate may change over time. However, the real-world acceptance rate was close to our results, which could diminish the effects from the above problems. The sustained period of the COVID-19 pandemic is hard to predict, and even though we have revealed the willingness to receive booster doses, it is difficult to provide a reference for following these doses.

## 5. Conclusions

Investigating the willingness to receive COVID-19 vaccination is important because it can identify the factors that may affect people’s likelihood of being vaccinated. However, it is not easy to have the results match real-world data. Here, we offer data on the willingness of elderly Taiwanese individuals to receive COVID-19 vaccination, and the coverage rate matches the actual situation. As in most countries, the elevated acceptance rate of the COVID-19 vaccine started after local outbreaks. However, the actual acceptance rate of the elderly population in Asian countries is lower than that in Europe or the U.S. This still poses a potential public health threat to such a fragile population while new variants of SARS-CoV-2 continue to appear. It is worth noting that older participants recruited from the outpatient department had a lower willingness to vaccinate than others. Healthcare providers should evaluate and give proper suggestions to these individuals. The long-term observation of the safety of COVID-19 vaccines is still lacking, and elderly people want to accept safer COVID-19 vaccines as existing vaccines affect their willingness to be vaccinated. Updating and educating elderly people on the latest information about the safety of available COVID-19 vaccines would help them make decisions.

## Figures and Tables

**Table 1:** Basic characteristics of the participants stratified by willingness.

Variables	Numbers	Positive willingness to Accept COVID-19 Vaccine ^1^	Negative willingness to Accept COVID-19 Vaccine	*p*-Value (COC) ^2^
	*N* = 957	*N* = 717	*N* = 240	
		74.9%	25.1%	
	*n*	*n* (%)	*n* (%)	
Sex				0.008 ** (0.086)
Male	446	352 (78.9)	94 (21.1)	
Female	511	365 (71.4)	146 (28.6)	
Age (years)				<0.001 *** (0.149)
60–69	377	301 (79.8)	76 (20.2)	
70–79	267	205 (76.8)	62 (23.2)	
80–89	216	155 (71.8)	61 (28.2)	
≥90	97	56 (57.7)	41 (42.3)	
Residential area				0.036 * (0.083)
Community	343	265 (77.3)	78 (22.7)	
Nursing home	337	236 (70.0)	101 (30.0)	
Out-patient department	277	216 (78.0)	61 (22.0)	
CCI				0.002 ** (0.099)
≤4	816	626 (76.7)	190 (23.3)	
≥5	141	91 (64.5)	50 (35.5)	
History of influenza vaccination				0.003 ** (0.111)
Yearly	535	406 (75.9)	129 (24.1)	
Not yearly	239	191 (79.9)	48 (20.1)	
Never	183	120 (65.6)	63 (34.4)	
Preference of origin of influenza vaccine				<0.001 *** (0.169)
Nil	799	573 (71.7)	226 (28.3)	
Taiwan	111	98 (88.3)	13 (11.7)	
Oversea	47	46 (97.9)	1 (2.1)	
History of pneumococcal vaccination				0.135 (0.084)
Yes	360	260 (72.2)	100 (17.8)	
Never	597	457 (76.5)	140 (23.5)	

^1^ We evaluated the willingness of accepting COVID-19 vaccines through participants responded with five options (very unlikely, somewhat unlikely, somewhat likely, very likely, and unsure). When a participant responded, “very likely” or “somewhat likely”, it was defined that the participant had a positive willingness of accepting COVID-19 vaccines. ^2^ The *p*-value was calculated using the chi-square test with effect size presented as coefficient of contingency. * *p*-value < 0.05; ** *p*-value < 0.01; *** *p*-value < 0.001. Abbreviation: COC, coefficient of contingency; CCI, Charlson Comorbidity Index.

**Table 2:** Awareness of the COVID-19 pandemic and attitudes toward receiving the COVID-19 vaccines.

Variables	Numbers	Positive willingness to Accept COVID-19 Vaccine ^1^	Negative Willingness to Accept COVID-19 Vaccine	*p*-Value (COC) ^2^
	*N* = 957	*N* = 717	*N* = 240	
		74.9%	25.1%	
	*n*	*n* (%)	*n* (%)	
Understanding the risk of being infected by SARS-CoV-2 ^3^				<0.001 *** (0.391)
Low	148	48 (32.4)	100 (67.6)	
Middle	92	68 (73.9)	24 (26.1)	
High	717	601 (83.8)	116 (16.2)	
Understanding the severity of the COVID-19 pandemic				<0.001 *** (0.350)
Low	134	47 (35.1)	87 (64.9)	
Middle	82	62 (75.6)	20 (24.4)	
High	741	608 (82.1)	133 (17.9)	
Understanding the effectiveness of COVID-19 vaccines				<0.001 *** (0.438)
Low	248	98 (39.5)	150 (60.5)	
Middle	126	102 (81.0)	24 (19.0)	
High	583	517 (88.7)	66 (11.3)	
Understanding the complications of COVID-19 vaccines				<0.001 *** (0.340)
Low	295	152 (51.5)	143 (48.5)	
Middle	134	110 (82.1)	24 (17.9)	
High	528	455 (86.2)	73 (13.8)	
Willingness to accept the COVID-19 vaccine for protecting others				<0.001 *** (0.433)
Low	281	123 (43.8)	158 (56.2)	
Middle	159	121 (76.1)	38 (23.9)	
High	516	472 (91.5)	44 (8.5)	
Safety of COVID-19 vaccines is a key factor for you to accept them				<0.001 *** (0.125)
Low	291	194 (66.7)	97 (33.3)	
Middle	124	96 (77.4)	28 (22.5)	
High	542	427 (78.8)	115 (21.2)	

^1^ We evaluated the willingness of accepting COVID-19 vaccines through participants responded with 5 options (very unlikely, somewhat unlikely, somewhat likely, very likely, and unsure). When a participant responded, “very likely” or “somewhat likely”, it was defined that the participant had a positive willingness of accepting COVID-19 vaccines. ^2^ The *p*-value was calculated using the chi-square test with effect size presented as coefficient of contingency. ^3^ Participants responded using a 7-point scale, and higher values indicate greater levels. When they responded with 1–3, 4, or 5–7 on the scale, the level of that construct was defined as low, middle, or high, respectively. *** *p*-value < 0.001. Abbreviation: COC, coefficient of contingency.

**Table 3:** Possible factors affecting the willingness of elderly individuals to accept the COVID-19 vaccines.

Variables	Numbers	Positive Willingness to Accept COVID-19 Vaccine ^1^	Negative Willingness to Accept COVID-19 Vaccine	*p*-Value (COC) ^2^
	*N* = 957	*N* = 717	*N* = 240	
		74.9%	25.1%	
	*n*	*n* (%)	*n* (%)	
Availability of vaccination sites ^3^				<0.001 *** (0.231)
Low	360	222 (61.7)	138 (38.3)	
Middle	159	133 (83.6)	26 (16.4)	
High	438	362 (82.6)	76 (17.4)	
Recommendation by family doctors				<0.001 *** (0.290)
Low	235	123 (52.3)	112 (47.7)	
Middle	154	118 (76.6)	36 (23.4)	
High	568	476 (83.8)	92 (16.2)	
Experiences from politicians				<0.001 *** (0.201)
Low	550	370 (67.3)	180 (22.7)	
Middle	171	147 (86.0)	24 (14.0)	
High	236	200 (84.7)	36 (15.3)	
Daily news from the media				<0.001 *** (0.202)
Low	481	342 (71.1)	139 (28.9)	
Middle	187	147 (78.6)	40 (21.4)	
High	289	228 (78.9)	61 (21.1)	

^1^ We evaluated the willingness of accepting COVID-19 vaccines through participants responded with five options (very unlikely, somewhat unlikely, somewhat likely, very likely, and unsure). When a participant responded, “very likely” or “somewhat likely”, it was defined that the participant had a positive willingness of accepting COVID-19 vaccines. ^2^ The *p*-value was calculated using the chi-square test with effect size presented as coefficient of contingency. ^3^ Participants responded using a 7-point scale, and higher values indicate greater levels. When they responded with 1–3, 4, or 5–7 on the scale, the level of that construct was defined as low, middle, or high, respectively. *** *p*-value < 0.001. Abbreviation: COC, coefficient of contingency.

**Table 4:** Knowledge of the COVID-19 pandemic and attitudes toward accepting booster doses of COVID-19 vaccines.

Variables	Numbers	Positive Willingness to Accept COVID-19 Vaccine ^1^	Negative Willingness to Accept COVID-19 Vaccine	*p*-Value (COC) ^2^
	*N* = 957	*N* = 717	*N* = 240	
		74.9%	25.1%	
	*n*	*n* (%)	*n* (%)	
Having information that SARS-CoV-2 could spread as influenza does yearly ^3^				<0.001 *** (0.330)
Low	350	193 (55.1)	157 (44.9)	
Middle	147	134 (91.2)	13 (8.8)	
High	460	390 (84.8)	70 (15.2)	
Willingness to accept a booster dose of COVID-19 vaccines in the future				<0.001 *** (0.451)
Low	329	147 (44.7)	182 (55.3)	
Middle	222	198 (89.2)	24 (10.8)	
High	406	372 (91.6)	34 (8.4)	

^1^ We evaluated the willingness of accepting COVID-19 vaccines through participants responded with five options (very unlikely, somewhat unlikely, somewhat likely, very likely, and unsure). When a participant responded, “very likely” or “somewhat likely”, it was defined that the participant had a positive willingness of accepting COVID-19 vaccines. ^2^ *p*-value was calculated using the chi-square test with effect size presented as coefficient of contingency. ^3^ Participants responded using a 7-point scale, and higher values indicate greater levels. When they responded with 1–3, 4, or 5–7 on the scale, the level of that construct was defined as low, middle, or high, respectively. *** *p*-value < 0.001. Abbreviation: COC, coefficient of contingency.

**Table 5:** Individual variables associated with positive willingness to accept the COVID-19 vaccines.

Variables		Model 1 ^1^			Model 2 ^2^			Model 3 ^3^			Model 4 ^4^		
	*n*	Odds Ratio	95% Confidence Interval	*p*-Value	Odds Ratio	95% Confidence INTERVAL	*p*-Value	Odds Ratio	95% Confidence Interval	*p*-Value	Odds Ratio	95% Confidence Interval	*p*-Value
Sex													
Male	446	1			1			1			1		
Female	511	0.71	(0.52–0.97)	0.033 *	0.76	(0.51–1.14)	0.188	0.81	(0.53–1.22)	0.305	0.79	(0.51–1.22)	0.281
Age (years)													
60–69	377	1			1			1			1		
70–79	267	0.87	(0.42–1.14)	0.500	1.52	(0.90–2.57)	0.116	1.54	(0.90–2.67)	0.115	1.78	(1.00–3.17)	0.050
80–89	216	0.69	(0.42–1.14)	0.145	1.75	(0.90–3.42)	0.099	1.78	(0.90–0.36)	0.099	2.17	(1.02–4.61)	0.045 *
≥90	97	0.39	(0.20–0.77)	0.007 **	0.97	(0.39–2.46)	0.973	1.00	(0.38–2.64)	0.650	1.20	(0.43–3.34)	0.735
Residential area													
Community	343	1			1			1			1		
Nursing home	337	0.90	(0.58–1.41)	0.652	2.08	(1.11–3.90)	0.023 *	1.64	(0.85–3.20)	0.142	1.48	(0.73–3.01)	0.276
Out-patient department	277	1.23	(0.82–1.86)	0.319	0.63	(0.36–1.10)	0.102	0.53	(0.29–0.97)	0.041 *	0.47	(0.25–0.91)	0.026 *
CCI													
≤4	816	1			1			1			1		
≥5	141	1.00	(0.59–1.72)	0.982	0.87	(0.42–1.79)	0.699	0.84	(0.39–1.79)	0.650	0.98	(0.44–2.18)	0.953
History of influenza vaccination													
Yearly	535	1			1			1			1		
Not yearly	239	0.81	(0.52–1.28)	0.366	0.97	(0.54–1.75)	0.928	1.02	(0.56–1.88)	0.942	1.05	(0.54–2.01)	0.890
Never	183	0.38	(0.24–0.61)	<0.001 ***	0.48	(0.22–0.88)	0.018 *	0.56	(0.30–1.06)	0.073	0.72	(0.37–1.41)	0.340
Preference of origin of influenza vaccine													
Nil	799	1			1			1			1		
Taiwan only	111	0.81	(0.52–1.28)	0.002 **	1.98	(0.90–4.37)	0.091	2.07	(0.93–4.65)	0.077	2.08	(0.88–4.91)	0.094
Oversea only	47	0.38	(0.24–0.61)	0.007 **	11.76	(1.43–97.16)	0.022 *	9.58	(1.13–80.96)	0.038 *	5.07	(0.60–42.96)	0.137
History of pneumococcal vaccination													
Yes	360	1			1			1			1		
Never	597	0.84	(0.56–1.24)	0.374	0.80	(0.47–1.39)	0.434	0.78	(0.45–1.37)	0.391	0.86	(0.47–1.59)	0.868
Understanding the risk of being infected by SARS-CoV-2 ^5^													
Low	148				1			1			1		
Middle	92				4.73	(2.07–10.79)	<0.001 ***	4.00	(1.70–9.45)	0.002 **	3.39	(1.34–8.60)	0.01 *
High	717				6.43	(3.25–12.73)	<0.001 ***	5.68	(2.79–11.56)	<0.001 ***	5.19	(2.40–11.25)	<0.001 ***
Understanding the severity of the COVID-19 pandemic													
Low	134				1			1			1		
Middle	82				1.62	(0.66–4.02)	0.295	1.48	(0.57–3.85)	0.424	1.14	(0.41–3.22)	0.801
High	741				0.74	(0.34–1.63)	0.457	0.61	(0.27–1.39)	0.237	0.54	(0.22–1.35)	0.187
Understanding the effectiveness of COVID-19 vaccines													
Low	248				1			1			1		
Middle	126				3.42	(1.80–6.49)	<0.001 ***	4.45	(2.27–8.72)	<0.001 ***	3.59	(1.77–7.31)	<0.001 ***
High	583				7.02	(4.03–12.24)	<0.001 ***	8.02	(4.45–14.46)	<0.001 ***	6.00	(3.22–11.17)	<0.001 ***
Understanding the complications of COVID-19 vaccines													
Low	295				1			1			1		
Middle	134				1.57	(0.80–3.11)	0.192	1.37	(0.69–2.75)	0.370	1.20	(0.57–2.52)	0.626
High	528				1.02	(0.57–1.80)	0.960	1.10	(0.60–2.01)	0.761	1.11	(0.58–2.13)	0.755
Willingness to accept the COVID-19 vaccine for protecting others													
Low	281				1			1			1		
Middle	159				2.75	(1.58–4.78)	<0.001 ***	2.61	(1.45–4.69)	0.001 **	2.46	(1.32–4.60)	0.005 **
High	516				12.93	(7.54–22.17)	<0.001 ***	10.60	(6.07–18.69)	<0.001 ***	8.56	(4.63–15.82)	<0.001 ***
Safety of COVID-19 vaccines is a key factor for you to accept them													
Low	291				1			1			1		
Middle	124				1.380	(0.69–2.56)	0.361	0.75	(0.34–1.65)	0.475	0.81	(0.35–1.86)	0.613
High	542				0.59	(0.36–0.96)	0.035 *	0.43	(0.25–0.75)	0.003 *	0.54	(0.30–0.99)	0.045 *
Availability of vaccination sites													
Low	360							1			1		
Middle	159							2.33	(1.09–4.99)	0.030 *	1.68	(0.74–3.82)	0.215
High	438							1.05	(0.60–1.82)	0.875 **	0.83	(0.45–1.51)	0.533
Recommendation by family doctors													
Low	235							1			1		
Middle	154							2.07	(1.01–4.23)	0.047 *	1.90	(0.88–4.09)	0.101
High	568							2.68	(1.50–4.78)	0.001 **	1.80	(0.96–3.38)	0.068
Experiences from politicians													
Low	248							1			1		
Middle	126							1.99	1.00–3.95	0.050	1.92	(0.92–4.00)	0.081
High	236							1.82	0.98–3.37	0.058	1.83	(0.94–3.55)	0.076
Daily news from the media													
Low	481							1			1		
Middle	187							0.83	0.45–1.56	0.568	0.77	(0.39–1.53)	0.459
High	289							0.74	0.43–1.29	0.291	0.79	(0.44–1.44)	0.440
Having information that SARS-CoV-2 could spread as influenza does yearly													
Low	350										1		
Middle	147										3.20	(1.46–6.99)	0.004 **
High	460										1.01	(0.57–1.76)	0.987
Willingness to accept a booster dose of COVID-19 vaccines in the future													
Low	329										1		
Middle	222										4.12	(2.24–7.58)	<0.001 ***
High	406										5.09	(2.66–9.75)	<0.001 ***

^1^ The independent variables were sex, age, CCI, residential area, history of influenza vaccination, preference of origin of influenza vaccine, and history of pneumococcal vaccination. ^2^ The independent variables were the factors in Model 1 plus understanding the risk of being infected by SARS-CoV-2, understanding the severity of the COVID-19 pandemic, understanding the effectiveness of COVID-19 vaccines, understanding the complications of COVID-19 vaccines, and willingness to accept the COVID-19 vaccine for protecting others, safety of COVID-19 vaccines is a key factor for you to accept them. ^3^ The independent variables were the factors in Model 2 plus the availability of vaccination sites, recommendation by family doctors, experiences from politician, and daily news form the media. ^4^ The independent variables were the factors in Model 3 plus having information that SARS-CoV-2 could spread as influenza does yearly and a willingness to accept a booster dose of COVID-19 vaccine in the future. ^5^ Participants responded using a 7-point scale, and higher values indicate greater levels. When they responded with 1–3, 4, or 5–7 on the scale, the level of that construct was defined as low, middle, or high, respectively. * *p*-value < 0.05; ** *p*-value < 0.01; *** *p*-value < 0.001. Abbreviation: CCI, Charlson Comorbidity Index score.

## Data Availability

The data will be available upon reasonable request to the corresponding authors.
